# Supplementary material for: Skin bacterial community differences among three species of co-occurring Ranid frogs
Source: PeerJ. 2023 Jul 14;11:e15556. doi: 10.7717/peerj.15556 (PMC10351513; doi:10.7717/peerj.15556)
Supplement: Supplemental Information 2 [file peerj-11-15556-s002.docx]

**Skin bacterial community differences among three species of co-occurring Ranid frogs**

**Supplemental Material**

Tadpole Indicator Species Analysis:

*Methods*: We used an indicator species analysis to determine which ASVs were significantly associated with bacterial community differences between tadpoles and adults. To do this, we used the *multipatt* function (func = *IndVal.g*, permutations = 999) in the *indicspecies* package in *R* (De Cáceres 2013). This analysis uses an ASV table to determine how strongly an ASV is associated with an amphibian species or life stage and computes a p-value.

*Results*: Lastly, based on the indicator species analysis, we found that 250 ASVs were significantly associated with *Lithobates* tadpoles. From these 250 ASVs, the majority belonged to phyla Proteobacteria (99 ASVs), Bacteroidetes (53), and Firmicutes (40). There was a substantial diversity of bacterial families, but some of the common families of the 250 ASVs were Ruminococcaceae (27), Burkholderiaceae (23), Acidobacteriaceae (17), and Acetobacteraceae (17).

Adult Amphibian Alpha and Beta Diversity Additional Analyses

*Methods*: We ran a PERMANOVA with only the adult amphibian species bacterial community data using species, subsite, infection status, and body condition as predictors. To calculate body condition we took residuals from a linear regression between snout-vent length and mass. We ran the PERMANOVA with both a Jaccard and Bray-Curtis dissimilarity matrix with 999 permutations each and in the *adonis2* function in the *vegan* R package. Additionally, we ran a GLM for both alpha diversity measurements, ASV richness and Shannon diversity, using the same predictors as in the PERMANOVA. We used a gamma distribution in the Shannon diversity GLM and a negative binomial distribution for the ASV richness GLM.

*Results*: We found that bacterial community structure differed across species (PERMANOVA Bray-Curtis: F_(2,47)_ = 3.21, R^2^ = 0.11388, p-value < 0.001; Jaccard: F_(2,47)_ = 2.4007, R^2^ = 0.09106, p-value < 0.001), site ( PERMANOVA Bray-Curtis: F_(4,47)_ = 1.9073, R^2^ = 0.13533, p-value < 0.001; Jaccard: F_(4,47)_ = 1.5373, R^2^ = 0.11662, p-value < 0.001), and body condition (PERMANOVA Bray-Curtis: F_(1,47)_ = 1.9849, R^2^ = 0.03521, p-value = 0.011; Jaccard: F_(1,47)_ = 1.5308, R^2^ = 0.02903, p-value = 0.018). Infection status was not a significant predictor of bacterial community structure (PERMANOVA Bray-Curtis: F_(1,47)_ = 1.3436, R^2^ = 0.02383, p-value = 0.146; Jaccard: F_(1,47)_ = 1.2451, R^2^ = 0.02362, p-value = 0.132). We found ASV richness was similar across sites, body condition, and infection status (all p-values > 0.05); and was only significantly lower in *L. palustris* (coefficient = -0.35706, SE = 0.18127, p-value <0.001) and *L. clamitans* (coefficient = -0.32204, SE = 0.20131, p-value = 0.0319). Shannon diversity did not differ across sites, infection status, or body condition (all p-values < 0.001). Only *L. sylvaticus* had a significantly great Shannon diversity measure coefficient = 0.115156, SE = 0.045198, p-value = 0.015).


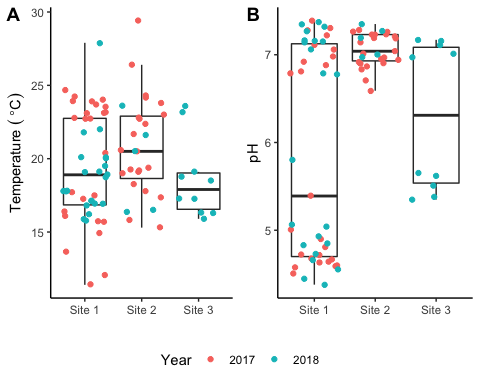


Supplemental Figure 1: We measured A) water pH and B) water temperature at all three sites amphibian were collected. Over the boxplot are the raw data values, colored by year.


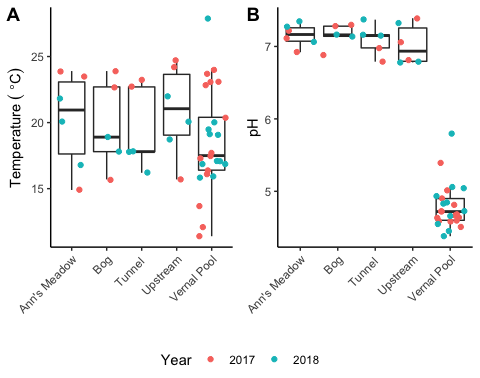


Supplemental Figure 2: Site 1 was broken into 5 subsites and at each subsite A) water temperature and B) pH were measured. Over the boxplot are the raw data values, colored by year.


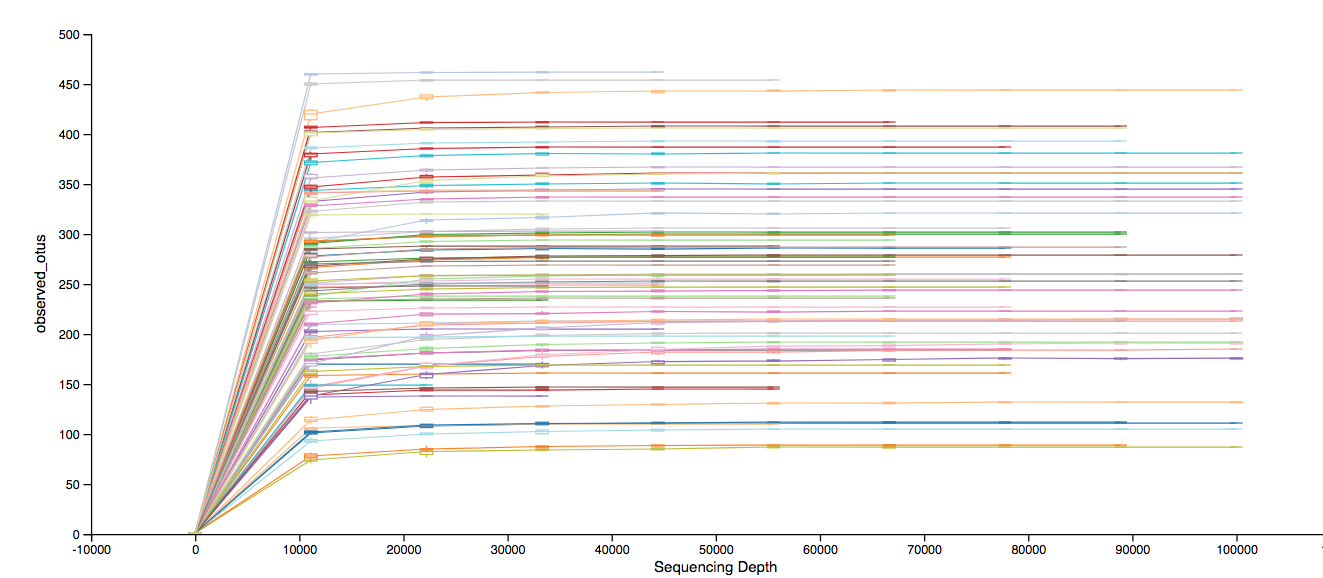


Sequencing Depth

Observed ASV

Supplemental Figure 3: The number of observed ASVs shown across sequencing depth. Based on this alpha rarefaction curve, the dataset was rarefied to 20000 reads.
